# Staying Together After Infidelity: An Exploration of the Decision‐Making Process of Recovery From the Perspective of the Injured Partner

**DOI:** 10.1111/jmft.70110

**Published:** 2025-12-28

**Authors:** Erica A. Mitchell, Kristina S. Brown, Jewel Spencer, Kayla Harris

**Affiliations:** ^1^ Department of Human Development and Family Studies Michigan State University 552 W. Circle Drive East Lansing Michigan USA; ^2^ Northwestern University Chicago Illinois USA; ^3^ Adler University Chicago Illinois USA

**Keywords:** affair recovery, infidelity, injured partner, qualitative

## Abstract

Infidelity impacts a significant number of people with potentially devastating consequences. Many people choose to stay with their partner following infidelity, but there is limited research on the decision‐making processes in affair recovery from the perspective of the injured partner. The current study explores this phenomenon through semi‐structured interviews with 11 injured (i.e., did not have an affair) partners who chose to remain with their partner after experiencing infidelity. Nine main themes were identified: (a) stance on infidelity, (b) sharing with others, (c) reasons to stay, (d) apology, (e) social support, (f) sexual intimacy expectations, (g) rebuilding trust, (h) therapy, and (i) other resources. These findings extend existing research on affair recovery by intentionally giving voice to the decision‐making experiences of injured partners in affair recovery. Considerations for clinicians working with individuals and couples, such as understanding the reasons to stay in the relationship and renegotiating relationship boundaries, are discussed.

## 1 Introduction

Infidelity impacts a significant number of relationships. Recent studies with large, representative samples across the United States have estimated 13%–40% of individuals engage in infidelity (e.g., Harrison 2024; O'Connor and Canevello 2019). One of the reasons for this variability is the various ways in which infidelity is defined. In the current study, we use the definition described by Blow and Hartnett (2005, pp. 191–192): a sexual and/or emotional act engaged in by one person within a committed relationship, where such an act occurs outside of the primary relationship and constitutes a breach of trust and/or violation of agreed upon norms (overt and covert) by one or both individuals in that relationship in relation to romantic/emotional or sexual exclusivity.

Following the discovery of infidelity, an injured partner (i.e., the person who did not have the affair) may experience a range of emotional and mental distress symptoms including depression, anxiety, and post‐traumatic stress disorder (Bird et al. 2007; Gordon et al. 2004). Infidelity‐based attachment trauma and the desire to be physically distant from one's partner are also reported (Apostolou et al. 2022; Warach and Josephs 2019). The perspective of injured partners is important to understand as the decision to stay together post infidelity often resides with this partner. Many times in fact there is pressure on the injured partner to forgive so that the relationship can move forward. This can be an extraordinary amount of responsibility, especially at a time when the injured partner may be overwhelmed with various emotions that can hinder the ability to make long‐term decisions about the relationship.

The literature also finds that relationship quality declines after infidelity and there is an increased likelihood of experiencing intimate partner violence (e.g., Kaighobadi et al. 2009; Previti and Amato 2004). More than 50% of injured partners will terminate their relationship following an affair (Allen and Atkins 2012), and infidelity is one of the most frequently cited reasons for divorce (Amato and Previti 2003). While the consequences of infidelity for injured partners have been documented in the literature, less is known about injured partners' decision‐making process and what influences the decision to stay in the relationship after infidelity. Given that the decision to stay often comes from the injured partner, a better understanding of this decision‐making process can positively benefit the therapeutic process by providing additional insight into this unique perspective with a focus on repair and recovery.

A better understanding of this unique perspective can also influence treatment models. Existing conceptual models of affair recovery have been developed using the perspectives of individuals who identify as injured or involved (i.e., the person who had the affair) partners. For example, Olson et al. (2002) identified a three‐stage model of experiences following infidelity: (a) roller coaster, (b) moratorium, and (c) rebuilding trust. Similarly, Bird and colleagues' (2007) findings revealed three phases to the healing process: (a) seeking expert assistance, (b) regaining a sense of control, increasing emotional openness, and rebuilding trust, and (c) establishing forgiveness. Abrahamson et al. (2012) identified four components of recovery: (a) motivation to stay together, (b) treasuring acts of kindness, (c) making meaning of the affair, and (d) utilizing social support. Efficacy studies to date have evidenced some improvements in individual and relationship outcomes for injured partners; however, the sample sizes have been very small and thus difficult to generalize the results (e.g., Atkins et al. 2010; Gordon et al. 2004). Existing research has also not helped us understand how the movement through each stage previously described is uniquely experienced by injured and involved partners. One prior study explored the recovery process from the perspectives of each partner within the same relationship. Both partners similarly valued (a) the importance of frequent communication, (b) an intentional approach to rebuilding safety and trust, and (c) the importance of forgiveness. Uniquely for injured partners, but not necessarily for involved partners, spending more time together was a mechanism for rebuilding trust and regaining a sense of closeness— essential to the middle stages in each of the presented models. For injured partners, the involved partner taking the lead on the recovery process and having the affair as an open topic for conversation was also essential (Mitchell et al. 2022). This request reinforces the purpose of the current study—the pressure and responsibility that is often placed on the injured partner. As such, these findings inspired the current study to continue to expand on this important work by deepening our understanding of the recovery process from the perspective of only injured partners who chose to remain in the current relationship.

### 1.1 The Current Study

Understanding affair recovery from the unique perspective of injured partners is essential for informing work with clients presenting to therapy following infidelity. While most of the research is from the perspectives of both injured and involved partners (e.g., Abrahamson et al. 2012; Bird et al. 2007; Olson et al. 2002), this study seeks to expand the current literature on the client experience of infidelity from the unique and sole perspective of the injured partner. Specifically, we seek to understand (1) the injured partner's decision to stay in the relationship and (2) the decision‐making processes in recovery. This research seeks to provide voice to this population's experience and identify factors involved in the decision to stay together and the affair recovery process. The focus is to provide clinicians with a deeper understanding of this under‐explored perspective in order to enhance clinical intervention.

## 2 Methods

The current study uses a qualitative phenomenological approach to meet the goal of deepening the understanding of the infidelity experience specifically from the injured partner's perspective. Through the structure of an interview and the opportunity for probing questions, findings are able to be better generalized to this treatment population. Several previous studies on infidelity have utilized this methodology (e.g., Abrahamson et al. 2012; Mitchell et al. 2021, 2022; Bird et al. 2007; Olson et al. 2002). Institutional Review Board approval was obtained from Adler University for the research. This research project took place as a part of a graduate‐level course. Students in the course were given the option to participate in the project or do an equivalent assignment to meet course requirements. All students opted to participate in the research project, though only one student (i.e., the third author) continued as part of the research team after the courses ended.

### 2.1 Participants

As the focus of this study is injured partners, the criteria for participation were (a) identity as the injured partner, (b) having maintained the relationship where the infidelity (as defined by Blow and Hartnett 2005 at the outset of this article) occurred, and (c) the discovery of infidelity must have occurred no sooner than 2 years prior. We included this timeframe to ensure that participants could reliably talk about the recovery process, while also reducing the likelihood of participants being triggered through participation in our study. Per IRB approval, participants were recruited through email and Facebook groups to which the students and the course instructor belonged—this did not include their personal profiles. The definition of infidelity used in the current study was included in the recruitment emails and postings.

During the semester the class was held, five participants responded. Two additional participants were recruited in the following months. After determining that data saturation had not yet been met, additional recruitment was done by the second author resulting in four additional respondents in the following year. As a result, there were a total of 11 responses to the recruitment posts and emails that met the inclusion criteria. Of the 11 participants, eight were recruited through Facebook, one participant through email, one through “a friend,” and one participant said “I was referred.” All 11 participants were interviewed. Subsequent analysis supported that saturation had been achieved (i.e., no new information was being shared in the interviews) and thus recruitment stopped.

As part of the informed consent, participants responded to open‐ended demographic questions. All injured partners identified as female with ten identifying as heterosexual or “straight” and one participant leaving sexual orientation blank. Discovery of the affair happened between 2 and 18 years prior to the interview with an average of 6 years (mode of 3 and median of 5). Participants also provided information about their partner (i.e., the involved partner). All involved partners were male with one participant also indicating female in the response as their subsequent relationship now includes herself as the injured partner, the involved partner, and the female “extra marital” partner in a polyamorous relationship. Nine participants identified their partners as heterosexual or “straight” and two participants left the sexual orientation of their partner blank. Seven of the respondents have children with four of those seven bringing children from previous partnerships into the current relationship. Participants were located in eight different states in the United States with one living in the country, three living in a small town, four in the suburbs, and three in the city. Individual annual incomes of the injured partner ranged from “$25,000 or less” to “$100,000 to $150,000,” with household annual incomes ranging from “$75,000 to $100,000” up to “more than $200,000.” Further demographics are provided in Table 1.

**Table 1:** Demographics of injured (*n* = 11) and involved partner (*n* = 11).

	Injured partner (participant)	Involved partner
Race		
Biracial (Black and White)	—	1
Black	—	1
Caucasian/White	9	7
Mexican/Mexican American	1	1
Blank	1	1
Spiritual/Religious Identity		
Catholic	2	1
Christian	5	5
Jewish	—	1
Methodist	1	—
None	2	3
Protestant	1	—
“Spiritual but not religious”	—	1
Education		
High School	1	1
Associates or “some college”	—	3
Technical College	1	—
Bachelor's Degree	—	5
Master's Degree	4	1
Doctorate (PsyD and PhD)	5	1
Employment Status		
Homemaker	2	—
Part‐time	1	—
Full‐time	8	10
Retired	—	1

*Note:* Injured partners ranged in age from 32 to 71 with a mean age of 45; involved partners ranged in age from 35 to 71 with a mean age of 47.

### 2.2 The Interview

The interview guide included an opening script and explorative questions that were developed within the graduate course by the 4 students and the faculty instructor (i.e., the second author). Questions were identified based on a review of the current literature and identification of current gaps in the understanding of the affair recovery process from the perspective of injured partners. Questions such as “What did you need most from your partner as you rebuilt your relationship?” and “What was most helpful to you in your recovery and decision to stay in the relationship?” as well as “What was least helpful to you in your recovery and decision to stay in the relationship?” focused specifically on what was needed in recovery from the sole perspective of the injured partner. Questions about recommendations for a therapist were also included. Participants were asked about the different stages of the decision‐making process:

1. What would you want a therapist to know about working with individuals who are deciding what to do after discovering their partner was unfaithful?
2. What would you want a therapist to know about working with a couple who hasn't decided whether or not to stay together after infidelity?
3. What would you want a therapist to know about working with couples who have decided to stay together after infidelity?

Interviewers were provided with only the email of the participant, referring to them by their selected pseudonym. A time to meet was mutually agreed upon via email between the participant and the interviewer as well as a reminder about maintaining privacy throughout the interview especially as all the interviews were conducted via HIPAA‐compliant Zoom provided by the second author's university. All interviews were conducted one‐on‐one by a student in the course or a research assistant of the second author with the participant.

Participants could opt to skip a question, limit their response, or turn off the camera if that was their preference. Each participant answered all questions to their own comfort level, and all participants chose to leave their cameras on. Interviews and transcriptions were completed by students and additional interviews were done by three research assistants of the second author following the class. Graduate students on the research team engaged in interviews and transcription, and the second author returned the transcripts to participants for member checking to confirm validity. No additional adjustments were subsequently made to the transcripts.

### 2.3 The Research Team

This study originated as a project from the second author's offering of an elective course on infidelity. The full research team included a collaborating content area faculty expert, a faculty instructor, and eight students. Two faculty, one doctoral student, and one master's student are authors on the current paper. We provide social location information about each of the authors to set the context of the research and analysis for the reader and as part of epoché throughout the qualitative process. The first author is a white, cisgender female and a licensed marriage and family therapist. She has worked clinically with couples who have experienced infidelity and has conducted other research, including qualitative studies, on this topic. The second author is a white, cisgender female, and licensed marriage and family therapist. She has taught specific elective courses on infidelity, looking at infidelity across identities and self‐of‐the‐therapist considerations. She has also worked clinically with relationships who stay together and separate as well as individuals who have either been the involved or injured partner or both. The third author is a black, cisgender, woman who works as a licensed marriage and family therapist in private practice and was a student in the class. She works clinically in conjoint and individual formats addressing challenges in the family system related to themes of repairing trust, communication, and the implications for changes in relationship boundaries. The fourth author is a white, disabled, cisgender, woman veteran who works as a licensed marriage and family therapist. She began participating in this project as a second year master's student for work study conducting interviews and coding for themes, and continued contributing as a doctoral student.

### 2.4 Data Analysis

With the goal of identifying patterns across the decision‐making processes of the participants, thematic analysis (Braun and Clarke 2006) in combination with epoché was used to ensure consistency across interpretation of the findings. Following the tenets of thematic analysis (Braun and Clarke 2006, 2021), the authors read through each of the interviews multiple times to familiarize themselves with the data. The first author and two student authors used a line‐by‐line coding process to capture the essence of participants' lived experiences. In regular meetings with the second author serving as internal auditor, the authors reviewed the 35 resulting codes combining them into themes across participants' perspectives of their own experiences. While we acknowledge the shared characteristics amongst our participants, for example, the fact that eight participants identified as mental health clinicians, we intentionally did not examine the data for similarities or differences based on this. We do suggest this as a future area of research in the discussion.

The authors engaged in reviewing, defining, and naming themes to determine significance. The authors used bracketing techniques such as journaling throughout the project and especially during data analysis to reduce the influence of individual bias on the team and the findings. Although the authors discussed their professional experiences learning about and working with infidelity, they did not discuss their personal experiences with infidelity. The team met regularly to work through the analysis and resolve differences in codes and themes. For example, the theme of *sexual intimacy expectations* was originally named “expectations,” but some members thought it was too broad. As a result, the team reviewed the coded transcripts again, determining that participants' descriptions were more focused on expectations around intimacy, and it was further revised to specify sexual intimacy. The external auditor independently coded each of the interviews and joined in research team meetings as the themes were refined and organized chronologically through participants' descriptions of the infidelity experience. When there was disagreement, the second author would weigh in as the internal auditor and the team would move to a consensus.

## 3 Findings

Each participant selected their own pseudonym, and the following is an introduction (in alphabetical order) including relationship status, infidelity disclosure information, and any history of infidelity within their relationship or family of origin.

Amy has been married for 6 years and found out about her husband's infidelity just over 2 years prior. She is unaware of any infidelity in her family of origin and her “husband's first marriage dissolved after his ex‐wife's infidelity.” Amy has not experienced infidelity in a previous relationship.

Crystal has been married for almost 18 years and found out about her husband's infidelity the first time 3 years prior and again a year later. After disclosure of the infidelity, Crystal and her husband decided together to invite the other woman into their relationship and family, and the three of them have been together for the last two‐and‐a‐half years. There has been no infidelity in any previous relationships for Crystal, in her husband's previous relationships, or her family of origin.

Emma has been married for 13 years and found out about her husband's infidelity 3 years ago. When asked about her family of origin she disclosed that before her parents divorced, “My mother cheated on my father several times. My father would always take her back, but he also may have cheated on her too.” In her husband's family, there has been no disclosure of infidelity, and her in‐laws have been together for 50 years. Emma experienced infidelity in her previous relationship where she discovered that her partner had been cheating on her throughout their relationship of almost 3 years.

Hope has been married for 12 years and discovered her husband's infidelity 3 years into their marriage. She has no firsthand experience with infidelity in her past. She found out about her mother's infidelity when she was an adult though she now has made connections to their arguing during her childhood, “Things were clicking!” Her husband's parents separated but still lived together for the last 7 years of their marriage. During that time, they both had relationships that were not disclosed until their children were adults, though Hope's husband remembers being suspicious.

Jade has been married for over 6 years and found out about her husband's infidelity less than 5 years prior. Though her parents are still married, she described that when she was 8 years old, her mother “sexually cheated on my father” and “shortly after, I was aware of an emotional relationship my father was having with [a] woman who lived in another state.” Her husband's parents were separated for 7 years and divorced when he was 14. “During those 7 years, each parent had other relationships, but did not expose those relationships to their children until they were older.” Jade shared that she had not experienced infidelity in a previous relationship.

KJ has been married to her second husband for over 20 years and found out about her husband's infidelity three years ago. KJ disclosed that she “had a two‐time sexual encounter at the end of my first marriage.” She shared that her “father had sex with prostitutes during his marriage to my mother” and that her mother‐in‐law “told me she observed her husband kissing another woman. She believes they might have had a relationship but he denied it.”

Laurie has been married for 39 years and discovered infidelity 18 years ago. She does not know of any infidelity that has occurred in her own family of origin or in any previous dating relationships. She disclosed that “my husband cheated on me after 21 years of marriage.” Both of her husband's parents had affairs during their marriage, including a long‐term relationship of his mother's that was “known by all in the family yet no one addressed it.”

Maggie has been married for 6 years, but together with her husband for 11 years. She found out about his infidelity “a little over 6 years ago.” She did not experience infidelity in previous relationships or in her family of origin. Maggie did share that when her brother‐in‐law passed away, his infidelity was discovered. Her husband was very angry especially because “he was all over me about what I did to you, but look at what he was doing, it was much worse.” Maggie shared that it “felt like almost another sense of betrayal when I found this out as [my brother‐in‐law] had been a major support when I found out about my husband's own infidelity.”

Maria's husband passed away 2 years ago, and she shared that the infidelity was discovered 6 years prior to that. Maria recollects that she “had sensed there was something wrong for probably 3 months prior.” She initially moved out but stated that “we worked hard to stay together.” She does not have any knowledge of infidelity on either side of their families and has not experienced infidelity in previous relationships.

May has been married for 15 years and the affair was discovered over 2 years prior. She described a past boyfriend who cheated on her, as well as reported that her mother had an affair in her relationship with her father. Her husband's mother was cheated on by both his father and later his stepfather. May shared, “I am still heartbroken—my husband says it was due to his childhood.”

Molly has been with her partner for 12 years discovering the second infidelity 9 years earlier. She described a family history of infidelity; her maternal grandfather cheated, which led to divorce, and her mother cheated on her father, which also led to divorce. Previously, Molly has had “several romantic relationships in which I was cheated on. … I did emotionally cheat … [but] … I made sure I broke up with him before I had sex with the new person. That ‘new person’ is my partner now.” Molly's partner's mother also had an affair, which led to divorce. With this shared history, she believed that they had “agreed these situations were wrong and ultimately destroyed families that we loved.”

**Figure 1:**
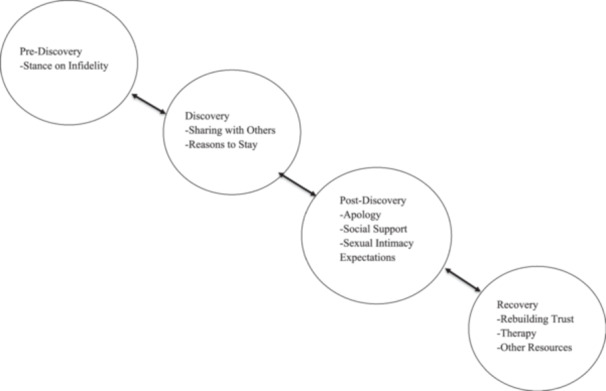
Decision to stay and decision‐making process of affair recovery for injured partner.

From these participants' interviews, nine main themes were identified: (a) stance on infidelity, (b) sharing with others, (c) reasons to stay, (d) apology, (e) social support, (f) sexual intimacy expectations, (g) rebuilding trust, (h) therapy, and (i) other resources. Using a thematic map (see Figure 1; Braun and Clarke 2006), the themes were organized chronologically through the relationship's timeline of the infidelity experience: (1) pre‐discovery: stance on infidelity (theme a), (2) discovery: sharing with others and reasons to stay (themes b and c), (3) post‐discovery: apology, social support, and sexual intimacy expectations (themes d, e, and f), and (4) recovery: rebuilding trust, therapy, and other resources (themes g, h, and i). We acknowledge that the timeline of experiences is not always linear, but merely a way to organize the identified themes as participants described them. For example, apology is not necessarily a single occurrence that happens before rebuilding trust, but rather may be an ongoing part of rebuilding trust in the relationship. In the tradition of qualitative research, direct quotes will be used as exemplars.

### 3.1 Pre‐Discovery: Stance on Infidelity

For most participants, the actual decisions they made after their partner's infidelity greatly differed from their prior stance or how they imagined they might respond. Prior to discovering the affair, participants described infidelity as generally unacceptable in their own relationship. Crystal shared, “I did not agree with it. Going behind someone's back is not okay.” Similarly Hope described herself as “a fiercely loyal person.” Despite these strong viewpoints on infidelity, participants did not explicitly discuss their viewpoint with their partner prior to disclosure of the affair. KJ stated, “So, my stance is then, anything that jeopardizes the relationship should not happen. But I don't know if I ever verbalized that.” All participants agreed in reflection that these beliefs were not overtly expressed within their relationships prior to the discovery of infidelity.

At the same time, participants acknowledged how common infidelity is despite, and because of, their own prior experiences with infidelity. These prior experiences included personal experience, through their partner's family, or in their own family of origin as described in their introductions. Jade said:

So, while I would like to say that if it ever happens to me, I would never stay with that person, it just isn't very realistic. I had seen it in so many relationships where some make it, and some don't make it. I think I just had an open stance on [infidelity].

### 3.2 Discovery of Infidelity

During the discovery phase, injured partners decided who to share the discovery of infidelity with, and ultimately identified reasons why they chose to stay in the current relationship. Here we describe participants' varied experiences with regards to discovering infidelity, in order to provide additional context to this phase of the affair recovery process. Some participants, like Amy, found out by accident, “while I had his phone, I got a message. Or he got a message I should say, basically saying ‘are you breaking up with me?‘” Other participants were suspicious of their partner's behavior and were intentionally seeking out information, as KJ shared:

I had been having a funny feeling like a, just an anxious feeling for quite a while. I started to notice that my husband talked a lot about this person and would interact with her a lot on social media. I was able to get into his email and it became quite apparent to me that he had been lying about a lot of their interactions.

Participants also described varying reactions to discovering infidelity. Amy described her reaction as “…very strong, like we don't do that. It was a shock.” Others said their initial reaction was to end the relationship. For example, Maggie explained, “When I first found out, when I was on my, I guess, I'll just call it, ‘my high horse,’ I was like I'm not going to stay with somebody who did this.” For Crystal, she needed that time as well as an option to leave (i.e., stay with her best friend) to make her decision, “after thinking about it, I had decided to stay at home and work through it.” The following themes are important to the decision‐making process for the injured partner to remain in the relationship.

#### 3.2.1 Sharing With Others

Participants described varied experiences regarding who, if anyone, to tell about the affair. Some participants chose to tell no one, and to the best of their knowledge their partner did not tell anyone either. Reasons for this ranged from faulty advice, fear of a lack of support in their ultimate decision, and shame around the changed view others would have of them and their relationship. Participants who chose to tell people were selective in their decisions, as KJ explained, “I didn't tell anyone in my family. I still haven't told anyone in my family.” Others described the process of sharing their experience as KJ continued, “I only told the one friend intentionally. There was another friend who called right in the middle of a horrible day, and she could tell something was wrong, and I just broke down and started crying.”

Some participants received a lot of support from the people they chose to share this with, as Crystal described, “My best friend and my mom were my biggest support systems.” When others found out about the infidelity, participants described some of the responses they received as generally unsupportive, especially during their decision‐making process post‐discovery. The range of responses received greatly impacted injured partners' experiences of social support during the recovery process; this is described further in a future subtheme of recovery, “social support.”

#### 3.2.2 Reasons to Stay

Participants identified various reasons for wanting to stay with their partner, including the length of their relationship and shared history. For KJ, this was a part of her decision to stay, “When you are married, there's an awful lot that binds you together, and tearing that apart is, is huge, right?” For others, their view of their partner was motivation to stay as they believed it would not happen again. For example, Emma described her partner as “always being trustworthy in the past.” Participants were willing to stay if they believed the affair was a single occurrence, as Crystal described, “I truly felt like it was a one‐time event…I felt like it wasn't going to happen again.”

Participants also described that their perceived strength of their relationship was a reason to stay. Hope described, “before all of this, we had not ever really had any major hiccup between us, we're just very compatible in a lot of ways.” The decision included weighing what the relationship was like previously and how the injured partner did not want the infidelity to define or shape their future relationship. Participants also described believing that the experience could be an opportunity to redefine their relationship moving forward. Additionally, the injured partner shared that they stayed because of the children they had with their partner. May said, “I've always thought when there's children you should try to make it work when possible. We have a child together. I do love him.” There was a belief that the negative impact of divorce on their family system would be greater in ending the relationship than in staying together and working on it.

### 3.3 Post‐Discovery of Infidelity

Following the initial phase of discovery and consistent with the models presented, apology, social support, and relationship expectations were all important parts of injured partners' lived experience of their decision‐making process. Participants were asked to reflect on the decisions they made in the short term after discovering the affair and how those experiences contributed to their choice to ultimately stay in the relationship.

#### 3.3.1 Apology

The involved partner's apology is something that most participants talked about as an important part of the affair recovery process. Apologies were described as “necessary but not sufficient” for many injured partner's healing. Many participants described their partner's apology as being critical after the discovery, as KJ explained:

For a long time, my husband couldn't apologize in a way that felt real. I think his shame was getting in the way of him really confronting how devastated I was by his behavior and so it was really hard for him for the longest time to really own that, but when he was able to do that, it was very healing. So, the apology was big.

Another participant, Crystal, described receiving apologies from both her husband and his affair partner: “Um, with my husband it was difficult because he has never been one to apologize or say he was wrong. And with her, she was very apologetic.”

While participants described an apology from their partner as helpful, they also acknowledged that an apology alone without any action or change in behavior was not sufficient. The participants agreed that words were not enough as they reflected on their decision to stay. Amy explained:

I don't know how many times he said sorry. And I'm sitting there going “sorry doesn't fix it.” So, apology, yes, but it was more the actions of remorse and the willingness to meet me where I needed to be. That played a bigger role.

Another participant, Maggie, echoed this sentiment:

I think in terms of apologies, yes it was nice that he apologized, but that wouldn't have been, it wasn't enough if he had just said, “Oh, I'm really sorry I did that. Okay, let's move on.” That wouldn't have worked. So, I think it was nice to hear the remorse and that you know this isn't something that was going to happen again.

Participants also acknowledged that apology wasn't always helpful because they didn't know if they could trust the sincerity. Participants' concerns centered around the fact that their partner had lied to them when having the affair. They struggled with how to believe that the apology was truthful, especially if not accompanied by remorse or action. This was a big factor in rebuilding trust as the relationship continued.

#### 3.3.2 Social Support

Participants described various degrees of social support that were present following the discovery of the affair, and how this influenced their decision to stay in their relationship. A common thread across participants' experiences of social support was the lack or presence of judgment. In other words, most participants described that whether or not they perceived judgment about the infidelity or the involved partner from those in their social network heavily influenced who they chose to reach out to, if anyone at all. This decision then directly influenced the support received by the injured partner. As described earlier, some participants had very limited social support due to their decision to not share about the affair with others. Repercussions of the affair including fear of judgment of themselves, their partner, and the decisions they would make about staying in their relationship were important considerations, as KJ described:

I didn't really have much of a support group. I think, because I felt such sorrow and shame about it and a sense of being stupid like I should have known, I should have done a lot of things differently. I didn't want people to think poorly of him.

Participants who did disclose the affair to others, or who were selective in whom they told, described receiving a lot of helpful support. Emma said, “I only talked to my one friend about it. I talked to her for quite some time. I did not have too much support in the thick of it besides my one friend.” Another participant, Maria, described the support received from multiple people in her life:

I think the support of my friends was really important, so all, all the people that I was able to talk to on the phone and would take my calls, mostly my friend [name]. She was willing to listen to me every day if I wanted to talk to her, and the people at work.

Participants all shared that even having just one supportive person was essential for their own recovery process as they provided a space of understanding and empathy without judgment. Further, participants found that support, as opposed to unsolicited advice, was more helpful from family and friends. Laurie said, “you shouldn't interject your own feelings into it … just offer support in that you are there for them … the least helpful [was] when people would tell me to end it.” One unique perspective regarding social support was provided by Molly. She shared that the lack of support was one of the reasons that they stayed together, “I think that probably played a huge part in as to why we ultimately got back together… he is my support system.”

#### 3.3.3 Sexual Intimacy Expectations

Following the discovery of infidelity, participants described the need to renegotiate expectations in their relationship, especially around sexual intimacy. For example, some participants experienced a lull in their sexual relationship, as Amy described, “Umm, we had none. I didn't want him to touch me.” While others, like Maggie, experienced improvements in the sexual relationship:

I think initially the sex was different. It was more like, it was more intimate if that makes sense. Where it wasn't just like, “Okay we got the job done. Whatever.” It was more like, just more of a connection. So, I think in the initial stages of coming back together, it helped to rebuild it in that way and just like increasing the connection and really kind of showing love through, you know, through physical intimacy.

Just as important as engaging in intimacy, participants described the dialog around intimacy as essential. For example, Amy shared, “It was never like ‘oh we need to start having sex again otherwise it's not going to work.’ It wasn't like that, it was definitely a gradual… ‘okay, I'm not as closed off to it right now’ [conversation]”. Though the focus was on romantic and physical intimacy, some participants acknowledged that intimacy in all parts of their relationships was essential to regaining connection moving forward. The opportunity for improvement of their relationship is not only recovery, but it is also a reason to stay.

### 3.4 Recovery

Once participants decided to stay and work on their relationship, they described the recovery process as important in confirming their decision to stay with their partner. The primary focus consistent across all participants was on rebuilding trust. Additionally, participants were asked about specific resources such as therapy, that contributed to their decision to stay.

#### 3.4.1 Rebuilding Trust

The ability to heal and regain trust in the relationship was described by participants as essential for recovery. Collectively, injured partners felt that it was not always easy to do in practice. Often the presumption is that the primary goal or focus is that the injured partner must learn to trust in their partner again. But, this is not sufficient alone. They also need to learn to trust themselves. For example, learning to trust that they will resist the urge to check in on their partner's every move, as Maria described:

I had to let go, you know? You have to let go, and just say, … if he says he's going to go play tennis then I'm going to trust that that's what he's doing. There was always this little voice in me about maybe I should follow him, or maybe I'm going to check in with him, maybe I'll call the club and see if he really goes there and stuff like that but I kind of resisted that because I didn't want to find myself in a position of having to be like an investigator.

Participants also shared a willingness to develop specific actions and negotiations (or “rules”) within their relationship to be helpful. For example, Emma described:

Reestablishing rules and comfort level with relationships with the opposite sex. I don't feel comfortable with him having a close relationship with somebody at work and needed to check in with his boundaries at work because he works mostly with women.

Involved partners playing an active role in helping injured partners rebuild this trust was also important. For example, Jade shared, “he told me I could look through his phone even though I didn't. My withholding from doing that was showing him that I trusted him and his giving me access was showing me that I could trust him.” Communication was also key to recovery. Molly said, “there were just a lot of long conversations, late night talking about what we wanted out of our relationship and where we saw this relationship going.”

The willingness of both partners to understand what led to the affair was described as an important part of deciding to stay in the relationship and rebuilding trust. Specifically, exploring the context of the relationship in which the affair occurred was an opportunity to understand what was happening and how to prevent affairs from occurring in the future. Maggie shared, “the fact that he took action was part of what made me say, ‘Okay I'm going to stay here’.” Another important part of communication for the injured partner was making sure that the involved partner was truly listening and hearing their concerns about the relationship. Crystal described this as an important part of rebuilding trust, “I would say that the first two things that come to mind is trust and being heard. I need to know that I can trust you and I need to know that you hear me.” Participants generally described this as a felt sense that the involved partner was being responsive to their concerns. This could be done both in the moment in the involved partner's response to what is shared and also outside of that moment as the injured partner is observing the interactions of the involved partner in response to the injured partner's concerns. Participants also talked about supportive resources contributing to their decision to stay in the relationship.

#### 3.4.2 Therapy

Participants were asked about attending therapy, and for those who attended, were asked about their experience of therapy as a part of the affair recovery process. Participants generally reported stark differences in their experiences of therapy, some finding it helpful while others finding it less so. Eight of the participants attended therapy; three attended couple therapy only and five attended couple and individual therapy. Of the participants who included therapy as part of their recovery process, three shared that their (involved) partner went to individual therapy as well. Half (*n* = 4) of the participants who went to couple therapy did not find it helpful. Laurie described feeling frustrated about her couple therapy experience, “at the very first meeting, he wanted to know what had happened. … then once we told him, he said, ok, now we won't talk about it again.” Whereas May reflected that the timing was off for her, “I think that people have to do individual therapy and restore themselves before they can come together and do some couples work.”

Other participants found therapy to be essential to the recovery process. Hope described her couple therapist as “so clutch” and said the therapist was essential to set boundaries and “rebuild that frame of safety for us.” This participant described the ways in which the therapist was supporting important facets of the affair recovery process, as reflected in the findings of the current study. For example, intentionally working with the couple on rebuilding safety as the clinician knows this is an important part of rebuilding trust. For those that did not go to therapy, one participant says they wished they had, in hindsight, gone to therapy, while others like Molly described a lack of access, “There's really not a lot of resources in our area. We live in a pretty rural community.”

#### 3.4.3 Other Resources

More than half (*n* = 6; 54%) of participants shared that they read various books during the process of recovery. May described this as a drive to read “anything that has to do with understanding him to understanding me.” Table 2 lists the books specifically mentioned by participants.

**Table 2:** Recommended books for infidelity recovery (in alphabetical order).

*Every Man′s Battle, Revised and Updated 20th Anniversary Edition: Winning the War on Sexual Temptation One Victory at a Time* by Stephen Arterburn & Fred Stoeker
*Healing Your Marriage When Trust Is Broken: Finding Forgiveness and Restoration* by Cindy Beall
*Permission to Feel*: The Power of Emotional Intelligence to Achieve Well‐Being and Success by Marc Brackett, PhD
Boundaries in Marriage: Understanding the Choices That Make or Break Loving Relationships by Dr. Henry Cloud & Dr. John Townsend.
*Every Woman′s Battle: Discovering God′s Plan for Sexual and Emotional Fulfillment* by Shannon Ethridge
*Transcending Post‐infidelity Stress Disorder (PISD): The Six Stages of Healing* by Dennis C. Ortman
*The State of Affairs: Rethinking Infidelity* by Esther Perel
After the Affair: Healing the Pain and Rebuilding Trust When a Partner Has Been Unfaithful, 2nd Edition by Janis Abrahms Spring, PhD*
How Can I Forgive You?: The Courage to Forgive, the Freedom Not To by Janis Abrahms Spring, PhD*
Not “Just Friends”: *Rebuilding Trust and Recovering Your Sanity After Infidelity* by Shirley Glass & Jean Coppock Staeheli

* Books with an asterisk were recommended by more than one participant.

Other resources, beyond therapy and books, were described by participants as an important part of their recovery process. For example, listening to *The Dr. Laura Show* with Dr. Laura Schlessinger, TED Talks by Esther Perel, and listening to Christian radio. May said that Christian radio “is very helpful to listen to it when I'm … struggling,” while other participants described the importance of spirituality in general throughout the recovery process. Additional resources were more unique to each participant. For example, Laurie shared both the importance of “dog therapy” (i.e., spending time with her dog) and her long‐time women's group (not specific to infidelity) through her church.

## 4 Discussion

This phenomenological study explored the lived experiences of injured partners who chose to stay in the relationship following the discovery of infidelity. While each person has an individualized experience of affair recovery, injured partners as a collective group have a unique set of experiences that have been underexplored in research as its own phenomenon. This study aimed to bring voice to their lived experiences with regards to deciding to stay in the current relationship and their decision‐making processes in recovery. The current findings inform working with clients seeking individual or couple therapy following an affair.

Participants described several factors that influenced their decision to stay in the current relationship—a long relationship history, shared experiences, having children together, belief in future fidelity (i.e., “one‐time event”), and the opportunity to create a better relationship moving forward. Participants in the current study were surprised by their ultimate decision to remain with their partner given their prior general stance that infidelity is unacceptable in relationships. Clinicians could normalize this when working with an injured partner who likewise is surprised by, and may be feeling uncertain about, their own decision to stay in the relationship. Many of the reasons identified for staying in the relationship though were ultimately more important to them than how they anticipated they would respond to discovering infidelity in their own relationship. For clinicians working with injured partners who want to stay in their relationship, understanding the reasons behind their desire to stay is crucial. While the decision to stay is often not a linear process, participants described important factors in the relationship in the immediate time following the discovery of the affair before the recovery process could begin.

Similarly to prior studies of affair recovery, our findings highlighted the importance of the involved partner's apology, ongoing communication, and the presence of social support as essential to rebuilding trust in the relationship (e.g., Abrahamson et al. 2012; Mitchell et al. 2021, 2022; Olson et al. 2002). Injured partners in the current study emphasized the need for apology accompanied *with* behavior change to really make an impact. Without witnessing behavior change, participants said they could not trust their partner's apology after the betrayal of the affair. This is an important finding to highlight, particularly for clinicians working with involved partners. The apology is much more than just the words “I'm sorry”—even if said countless times—it is taking action or changing one's behavior to offer that reassurance that infidelity will not occur again the future. Clinicians can intentionally explore what apology has looked like, if at all, up to the point that the client(s) come to their office. Further, a structured and safe space for apology to occur can be facilitated by the clinician in the therapy office.

While ongoing communication is essential for recovery (e.g., Mitchell et al. 2022; Olson et al. 2002), this communication will look different for every relationship. Setting expectations and boundaries specifically around communication about the affair is essential. For example, immediately following the affair discovery, an injured partner may need to constantly talk about the affair. Or partners who seek therapy early in the recovery process may choose to only talk about the affair during therapy sessions. Additionally, partners with children may agree not to talk about the affair where their children could overhear their conversation. Clinicians should actively facilitate these conversations in therapy to help their clients decide the specific communication boundaries and expectations that will be most appropriate for them and their relationship.

Another important communication boundary to set is with regards to who, if anyone, the partners will talk with about the affair. Consistent with our findings, prior research found social support to be an important factor in affair recovery (e.g., Abrahamson et al. 2012), and the participants in the current study emphasized the importance of *quality* social support. While there may be a tendency for injured partners to disclose the infidelity to others out of anger or a desire to hurt the involved partner, this can have consequences for the relationship later (e.g., low relationship quality between the person they told and the involved partner). Other injured partners may feel an immense amount of shame around the affair and thus choose not to disclose it to anyone. This could be related to participants' pre‐discovery perceptions of infidelity as being unacceptable and thus difficult to share with others that they had experienced this and were choosing, or at least thinking about, staying with their partner. Similarly to facilitating dialog around communication boundaries in general, this is another tricky piece of the recovery process that clinicians should take an active role in. For example, discussing with injured partners the list of people they are contemplating sharing this with, and what are the potential benefits and drawbacks of sharing with each person on their list.

From the unique perspective of injured partners in the current study, participants described vastly different experiences with regards to deciding who to tell, and the type of response and support they received from those whom they told about the affair. As part of the decision‐making process, injured partners are encouraged to think about what they need and who will respond in a helpful way. For example, some injured partners identified that someone telling them to leave their partner was not helpful for them or their recovery process. As a part of disclosing the affair, it is okay for injured partners to let their support people know what they need, and to acknowledge that what they need will likely change over time. Clinicians can support injured partners in developing their skills to ask for what they need from their support system. Another important consideration for partners with children is the decision of what, if anything, to tell their children about the affair. This is a complex decision with many factors to consider (e.g., age of children; potential change in family living circumstances, such as relocation), and therapists can support clients in making this decision. Focusing on this process was beyond the purview of the current study.

Participants acknowledged a lack of explicit conversation with their partner about their expectations of boundaries in their relationship prior to the affair, but that after the discovery participants recognized this conversation as crucial. Infidelity or the “betrayal of relationship boundaries” looks different from relationship to relationship, and thus the boundaries of the relationship should be clearly defined and agreed upon by (all) members of that relationship. Couples are encouraged to explicitly discuss their boundaries and develop a relationship “contract” that defines fidelity and infidelity as well as monogamy and non‐monogamy (Brown et al. 2023). Further, couples can schedule regular check‐ins to review the relationship contract and make changes as needed. For example, as the relationship evolves over time and the couple experiences life transitions (e.g., having children, new job, relocation), the boundaries of the relationship contract may need to change as well. For couples who experience infidelity, this contract may need to be renegotiated more frequently to ensure safety—particularly for the injured partner ‐ in the recovery process. For example, something that was previously acceptable in the relationship, such as a one‐on‐one meeting with a co‐worker, may no longer be after an affair or at least for some period of time until trust can be re‐established. As injured partners identify what they need to feel safe during this tumultuous time, the newly established boundaries may impact other aspects of the couple's life—good and bad—that will need to be renegotiated.

Prior research has included seeking expert assistance in models of affair recovery (e.g., Bird et al. 2007). Injured partners in the current study reported varied experiences with regards to engaging in therapy. While therapy can be helpful for affair recovery, many couple therapists find it difficult to work with infidelity (e.g., Barraca and Polanski 2021), often due to a lack of specific training in working with this complex presenting problem. Thus, therapists are encouraged to pursue education or additional training in how to effectively work with clients presenting with infidelity concerns. For example, the Couples and Intimate Relationships Topical Interest Network through the American Association for Marriage and Family Therapy (AAMFT) hosts webinars and trainings on various aspects related to intimate relationships, including infidelity.

It is also important that clinicians be aware of self‐of‐the‐therapist issues that may be triggered by working with infidelity and should seek supervision or peer support as needed. Further, infidelity is a traumatic event and thus therapists who work with clients who have experienced infidelity should prioritize their own self‐care, too. Even though couple therapy can be helpful for affair recovery, therapy may also be inaccessible for many clients in need due to various barriers (e.g., location, cost, time, childcare). For clients who live in areas (e.g., rural) with limited access to couple therapists (and even more so for therapists who are trained in working with infidelity), telehealth may offer a viable option to get support, especially as this has become a more widely available option.

For some injured partners, online or community‐based resources may be helpful for their recovery process too. Following the discovery of an affair, injured partners are often desperate for help. The difficulty is in finding “good” help and resources that will support the recovery process and not do further damage to their well‐being or to the relationship. We advocate for the development of an affair recovery clearinghouse—a collection of resources that have been recommended by injured partners and vetted by professionals with extensive training in working with affair recovery. An example of this is Table 2, which lists the books recommended by our participants which would then be vetted by experts. These resources could also be helpful for couple therapists working with infidelity, as these types of support can both enhance their own knowledge and understanding of infidelity and enrich the work being done in therapy. The existence of a clearing house could reduce the burden on therapists to vet resources themselves and provide a ready‐made library to choose from to best meet their clients' needs.

## 5 Limitations

Unique to this study was the shared professional identity of the majority (*n* = 8) of the 11 participants as mental health practitioners (e.g., LMFTs and Psychologists). Important considerations include not only the identities of the full research team who are also all systemic therapists, but also recruitment methods. In reviewing the sources of recruitment, only three participants came from Facebook groups that were specifically for clinicians, although the three responses that just said “Facebook” could be a specifically related group as could the other sources of referral. Though there may have been a recruitment bias towards mental health practitioners because of these factors, also of interest would be understanding why a participant made the decision to participate in the current study. For example, there may be a “calling” from clinicians to share their experiences that parallel those that are presented by clients. The authors discussed the option to separate out these eight participants, but as feminist researchers felt a responsibility to share the experience of all the participants. Instead, we identify this as a recommendation for future research.

Further, the majority of the participants were white (*n* = 9) and at the time of participation, identified as heterosexual (*n* = 10). We further note that not all participants reported their own (*n* = 10) or their partner's (*n* = 9) sexual orientation. A revised recruitment strategy to respond to these limitations could reach a more diverse sample of participants and their partners. Though findings are beneficial to the clinical work done with this population, the ability to generalize is limited as differing identities and intersections of identities are not well represented.

### 5.1 Future Research

There is more to the current data set that warrants additional exploration. Although it could be considered a limitation to this study that 8 of the 11 respondents are clinicians, it is also an area for future research, especially as 5 of them (63%) utilized therapy themselves. Interestingly, none of the participants expressed using therapy specifically to aid in deciding about the future of the relationship, but that it was one tool to aid in recovery. Clinicians may not feel a need to engage in their own therapy in the same way as people without clinical training as they have their own set of knowledge and professional experience to rely on as well as social networks amongst professional colleagues (e.g., Jade described her psychology classmates as a source of support). Barriers to accessing therapy were also mentioned, and a deeper exploration of the decision process to engage therapy following the discovery of infidelity could be an area of future research. A unique narrative from this research is Crystal's experience where there was a shared decision to invite the affair partner into the relationship. A better understanding of this decision‐making process can provide more information about adapting relationship structures, e.g., polyamory or consensual nonmonogamy, as a way of navigating the recovery process.

## 6 Conclusion

The injured partner's decision‐making process to stay in the current relationship is complex. While there is a heavy focus on specific needs of the injured partner, it is important to balance this with the responsibility for making the decision to maintain the relationship. A successful recovery process ultimately takes intentional effort from both injured and involved partners to make the relationship work. Barraca and Polanski (2021) maintain that infidelity is “one of the most damaging and difficult problems to treat in couple therapy” (p. 910) citing far reaching impact to everyone impacted by the infidelity. Continued exploration of not only the risk factors, but also a deeper understanding of the recovery process and unique perspectives (e.g., injured vs. involved partners), can further contribute to the literature on infidelity and inform clinical work with this complex presenting problem.

## Supporting information

JMFT R&R_Supplemental Material 1.
