# Supplementary material for: Staying Together After Infidelity: An Exploration of the Decision‐Making Process of Recovery From the Perspective of the Injured Partner
Source: J Marital Fam Ther. 2025 Dec 28;52(1):e70110. doi: 10.1111/jmft.70110 (PMC12745057; doi:10.1111/jmft.70110)
Supplement: Supplementary file 1 — JMFT R&R_Supplemental Material 1. [file JMFT-52-0-s001.docx]

**Supplemental Material 1.** Semi-structured interview guide

1. Tell me how you learned about the infidelity.
2. Prior to disclosure, what was your stance on infidelity?
3. Who else knew about the infidelity?
   1. How did their knowledge of the infidelity impact your relationship with them?
4. Tell me about your decision to stay in the relationship?
5. Tell me about your support system in recovery after disclosure? For example, family, friends, faith, etc.
   1. Was the support individual to you, your partner, and/or to your relationship?
6. Did you ever consider ending the relationship?
   1. What characteristics do you attribute to yourself that contributed to your decision to stay in the relationship?
   2. What characteristics did/does your partner hold that contributed to your decision to stay in the relationship?
7. What did you do to begin building safety and trust in your relationship?
8. What did you do to regain feelings of closeness in your relationship?
9. Were there times that you were reminded of the infidelity during your recovery?
   1. If yes, how did you manage that?
10. What did you need most from your partner as you rebuilt your relationship?
    1. Was your partner able and available to meet those needs?
11. What was most helpful to you in your recovery and decision to stay in the relationship?
12. What was least helpful to you in your recovery and decision to stay in the relationship?
13. What role did apology and forgiveness play in your recovery?
14. What impact did the infidelity have on your sexual relationship?
    1. What role did your sexual relationship play in rebuilding your relationship?
15. Did you attend therapy?
    1. If yes, what kind of therapy?
16. Did you read books you found helpful?
17. What professionals should know:
    1. What would you want a therapist to know about working with individuals who are deciding what to do after discovering their partner was unfaithful?
    2. What would you want a therapist to know about working with a couple who hasn’t decided whether or not to stay together after infidelity?
    3. What would you want a therapist to know about working with couples who have decided to stay together after infidelity?
18. What would you say to a person who was considering having an affair?
